# Comment on “Time-Varying Exposure to Element Mixtures and Children’s Cognition at 5 Years of Age: Findings from the New Hampshire Birth Cohort Study”

**DOI:** 10.1021/EHP.6c01150

**Published:** 2026-09-07

**Authors:** Cai Zhang, Yu Lin

**Affiliations:** † Department of Health and Kinesiology, 14589 University of Illinois Urbana-Champaign, Champaign, Illinois 61820, United States; ‡ Xiamen Medical College, Xiamen 361023, China

We commend Notario Barandiaran et al.^1^ for their thorough analysis of time-varying metal mixtures and child cognition in the New Hampshire Birth Cohort Study. Their application of lagged kernel machine regression to repeated biomarkers across gestation and infancy is a valuable contribution to a difficult problem, and the inverse association they report for arsenic is compelling. However, two aspects of the exposure assessment and interpretation warrant further consideration.

First, although the authors interpret their estimates as distinct windows of susceptibility, the toenail biomarker may not fully support this resolution. As they note, a toenail integrates the 6 to 12 months required for the plate to grow out,^2^ so the maternal samples at 24–28 weeks of gestation and at 6 weeks postpartum likely reflect overlapping segments of a single, slowly accumulating record; consistent with this, the authors report that within-mother cross-window correlations exceed the mother-infant correlations. The fused-lasso penalty in the estimator further shrinks differences between adjacent windows.^3^ The authors could strengthen their window-specific inferences by reporting the full cross-window correlation matrix and by considering whether the two maternal samples are better modeled as a single integrated prenatal exposure, presenting the remaining contrasts as hypothesis-generating.

Second, the associations reported for essential metals may warrant a more cautious interpretation. The authors acknowledge that toenail lead and copper do not correlate well with internal dose, and lead was in fact positively associated with two cognitive indices, a pattern they could not readily explain. Because toenail copper and manganese also co-vary with maternal nutrition and socioeconomic position, both strong predictors of child cognition, the positive associations for these essential metals may partly reflect residual confounding rather than neuroprotection. The authors could temper the suggestion that copper and manganese confer beneficial effects warranting future interventions, or probe it with negative-control^4^ or nutrition-adjusted analyses.

Finally, given the many metal, window, sex, and outcome combinations examined relative to the sample size, some findings, such as the large manganese-by-sex interaction, may be unstable, and a formal multiplicity adjustment would help distinguish robust signals. These refinements would strengthen an already valuable contribution to our understanding of early-life metal exposure and child cognition.
